# Whole-transcriptome splicing profiling of E7.5 mouse primary germ layers reveals frequent alternative promoter usage during mouse early embryogenesis

**DOI:** 10.1242/bio.032508

**Published:** 2018-03-15

**Authors:** Xukun Lu, Zhen-Ao Zhao, Xiaoqing Wang, Xiaoxin Zhang, Yanhua Zhai, Wenbo Deng, Zhaohong Yi, Lei Li

**Affiliations:** 1 State Key Laboratory of Stem Cell and Reproductive Biology, Institute of Zoology, Chinese Academy of Sciences, Beijing 100101, China; 2 University of Chinese Academy of Sciences, Beijing 100049, China; 3 Division of Reproductive Sciences, Cincinnati Children's Hospital Medical, Cincinnati, OH 45229, USA; 4 Key Laboratory of Urban Agriculture (North) of Ministry of Agriculture, College of Biological Science and Engineering, Beijing University of Agriculture, Beijing 102206, China

**Keywords:** Alternative promoter, Alternative splicing, Germ layer specification, Gastrulation, Mouse embryogenesis

## Abstract

Alternative splicing (AS) and alternative promoter (AP) usage expand the repertories of mammalian transcriptome profiles and thus diversify gene functions. However, our knowledge about the extent and functions of AS and AP usage in mouse early embryogenesis remains elusive. Here, by performing whole-transcriptome splicing profiling with high-throughput next generation sequencing, we report that AS extensively occurs in embryonic day (E) 7.5 mouse primary germ layers, and may be involved in multiple developmental processes. In addition, numerous RNA splicing factors are differentially expressed and alternatively spliced across the three germ layers, implying the potential importance of AS machinery in shaping early embryogenesis. Notably, AP usage is remarkably frequent at this stage, accounting for more than one quarter (430/1,648) of the total significantly different AS events. Genes generating the 430 AP events participate in numerous biological processes, and include important regulators essential for mouse early embryogenesis, suggesting that AP usage is widely used and might be relevant to mouse germ layer specification. Our data underline the potential significance of AP usage in mouse gastrulation, providing a rich data source and opening another dimension for understanding the regulatory mechanisms of mammalian early development.

## Abstract (precis)

**Summary:** This study seeks to capture the alternative splicing landscape during mouse gastrulation, underlining the potential importance of alternative promoter usage in mammalian early embryogenesis.

## INTRODUCTION

Sophisticated spatial-temporal regulation of gene expression is a prerequisite for proper mammalian development. Transcription factors and epigenetic regulators of the transcriptional-level control are generally at the focus of attention (Lee and Young, 2000). Transgenic and gene-targeted mice models have contributed greatly to elucidation of the functions and molecular mechanisms of these regulators (Bouabe and Okkenhaug, 2013). However, we are far from fully understanding the mechanisms underlying developmental programs. Recently, regulation of gene expression during development by alternative splicing (AS) and alternative promoter (AP) usage has begun to be brought to the fore as important candidate machineries to regulate multiple biological processes (Baralle and Giudice, 2017).

As an important dimension of gene expression regulation at the post-transcriptional level, AS greatly expands the mRNA and protein structural complexity, and so diversifies their functions of specific genes (Irimia and Blencowe, 2012; Keren et al., 2010). High-throughput RNA sequencing reveals that over 90% of human genes undergo AS (Pan et al., 2008), which is also reported to occur frequently during mouse early embryogenesis from embryonic day (E) 8.5 to E11.5 (Revil et al., 2010). Apart from AS, AP usage, which produces transcripts from different transcription start sites (TSSs), was recognized as another important mechanism to create diversity and flexibility of gene expression (Ayoubi and VanDeVen, 1996). About half of human and mouse genes produce diverse mRNA isoforms by using APs (Davuluri et al., 2008). Selective use of APs generates transcripts that might differ in their 5′ untranslated regions or coding sequences and thus alters the abundance, subcellular localization or activities of their protein products, eliciting cell-, tissue- and developmental-stage-specific functional patterns (Ayoubi and VanDeVen, 1996; Davuluri et al., 2008).

AS and AP usage are often neglected in traditional studies of gene function. However, encouraging findings have begun to unravel the biological implications of these machineries in development. For example, the transcription factor MEF2D is alternatively spliced during muscle differentiation to generate a muscle-specific isoform, MEF2Dα2, by using the mutually exclusive forth exon rather than the third exon, which is incorporated by the ubiquitously expressed isoform Mef2Dα1 (Martin et al., 1994). Although both isoforms bind to a set of overlapping genes, exon switching allows Mef2Dα2 to escape the otherwise inhibitory phosphorylation by protein kinase A, thus specifically activating late muscle development related genes (Sebastian et al., 2013). In mouse oocytes, use of an alternative intronic MT-C retrotransposon promoter of the *Dicer* gene locus generates an oocyte-specific isoform, DICER^o^, that is indispensable for mouse oocyte development (Flemr et al., 2013). DICER^o^ lacks the N-terminal DExD helicase domain and has higher endoribonuclease activity compared to the full-length somatic DICER (DICER^S^) and is the dominant isoform in oocytes to control the endogenous RNAi pathway. Additionally, *Runx1*, which encodes an essential transcription factor regulating hematopoiesis, is expressed from two APs, the proximal P2 and the distal P1 promoter (Ghozi et al., 1996), the activities of which are spatio-temporally modulated during embryogenesis (Webber et al., 2013). The activity of the proximal P2 is required for primitive hematopoiesis, while the distal P1 prevails in definitive hematopoietic stem cells (Pozner et al., 2007). Moreover, a recent work revealed that ubiquitously expressed genes could exert cell-specific functions via AP usage (Feng et al., 2016). Nonetheless, our knowledge about the functions and mechanisms of AS and AP usage, especially in mouse early embryonic development, is very limited.

Specification of the three primary germ layers during gastrulation is a fundamental phase in most animals to establish the body plan (Peng et al., 2016; Solnica-Krezel and Sepich, 2012; Tam and Behringer, 1997). In the present study, we explore the involvement of AS in regulation of mouse early development by mRNA profiling of the three embryonic germ layers of E7.5 mouse embryos. Our results show that AS is extensively utilized in the process of mouse gastrulation, which may be attributed to the finely modulated expression of splicing factors across different germ layers. Remarkably, we find that AP usage is prevalent at this stage, possibly contributing to gene expression regulation in mouse early development. Our study provides new insights into the control of mouse development with respect to how AS and AP usage mechanisms function during mouse early embryogenesis.

## RESULTS

### Whole-transcriptome RNA-seq profiling of E7.5 mouse primary embryonic germ layers

Next generation RNA sequencing is a powerful tool to capture the whole transcriptome dynamics of specific cell lines or tissues for downstream transcripts-based analysis, such as gene expression or AS profiling (Wang et al., 2009). Recent development of single-cell techniques enables investigation of genome-wide gene expression patterns at single cell level or with trace amount of input RNAs, such as materials from mammalian early embryos. However, technical limitations, including low coverage and sensitivity, 3′ bias, and higher technical noise, make it difficult to analyze isoform variations (Brennecke et al., 2013; Saliba et al., 2014; Stegle et al., 2015). To explore the possible involvement of AS in regulation of mammalian early development and identify potential functional AS events, we isolated the embryonic endoderm, mesoderm and epiblast (neuroectoderm and primitive streak) from E7.5 mouse embryos by micromanipulation for conventional bulk RNA sequencing (Fig. S1A). A total of 6.60 μg, 7.74 μg and 7.70 μg RNA of endoderm, mesoderm and epiblast, respectively, from hundreds of E7.5 mouse embryos were obtained (Fig. S1B). RNA integrity number (RIN) and the 28S to 18S rRNA ratio showed that the quality and integrity of the isolated RNAs were qualified for RNA sequencing (Fig. S1B).

We next examined the dissection efficiency using cell lineage-specific genes, including endoderm markers *Sox17* and *Foxa2*, mesoderm markers *Foxf1* and *Flk1*, and epiblast markers *Sox2* and *Pax6*. Quantitative PCR (qPCR) showed that the marker genes of each germ layer were highly or exclusively expressed in corresponding samples, indicating efficient separation of germ layers and little inter-sample contamination (Ma et al., 2016). Then, whole transcriptome profiling was performed. A total of 49,744,660, 49,753,030, 51,036,608 clean reads and 41,988,506 (84.4%), 42,140,982 (84.7%), 43,225,419 (84.7%) mapped reads were generated for the endoderm, mesoderm and epiblast, respectively (Table S2). RNA-seq data analysis revealed that a total of 17,633 genes were detected from the three germ layers.

**Fig. 1.**
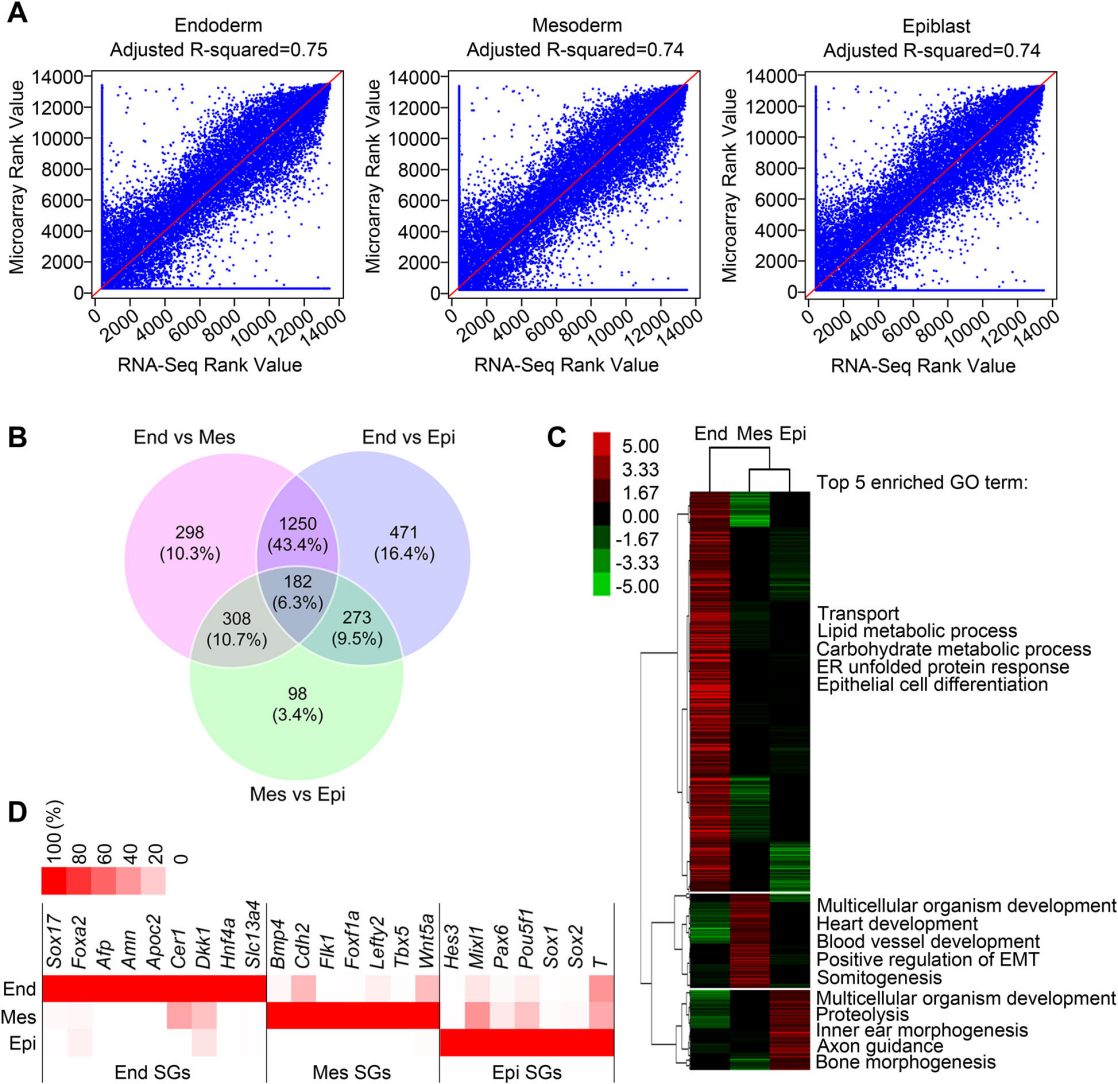
**Whole-transcriptome profiling of E7.5 mouse primary germ layers.** (A) Scatter plot showing the comparison result of the RNA-seq data with an independent Microarray analysis. (B) Venn diagram of the number of the differentially expressed genes in the three germ layers (at least one RPKM>5, Fold Change>2, FDR<0.001). (C) The heat map of genes highly expressed in each germ layer. The top five enriched GO terms of each cluster of genes are shown on the right. (D) The relative enrichment of representative germ layer signature genes (SGs) in each germ layer determined using RNA-seq data. Data are expressed as the percentage of each gene's RPKM to the maximum one in the three germ layers. End, endoderm; Mes, mesoderm; Epi, epiblast. See also Fig. S1.

To evaluate the quality of our RNA-seq data, we compared the RNA-seq data with an independent Microarray analysis of E7.5 mouse embryonic germ layers performed in our lab. We used the same set of genes detected by both technologies in each germ layer, and the consistency was evaluated based on each gene's rank expression value using Pearson Correlation Coefficient (PCC). The result showed that the gene expression obtained from RNA-seq data was in good accordance with that measured in the Microarray analysis (Fig. 1A). With the criteria of at least one RPKM>5, Fold Change>2 and FDR<0.001, 2,880 genes were found significantly differentially expressed in the three germ layers (Fig. 1B; Fig. S1C). Among the 2,880 genes, 1,019, 243 and 206 genes were highly expressed in endoderm, mesoderm and epiblast, respectively (Fig. 1C; Fig. S1D). Gene ontology (GO) analysis with Database for Annotation, Visualization and Integrated Discovery (DAVID) revealed that these dominant genes participated in specific biological processes relevant to each germ layer (Fig. 1C). The germ layer-specific signature genes (SGs) were specially enriched in each corresponding germ layer (Fig. 1D). All these data indicate the good quality and reliability of the whole transcriptome RNA-seq data, which could be used for AS analysis.

### Alternative splicing signature of the embryonic germ layers

**Fig. 2.**
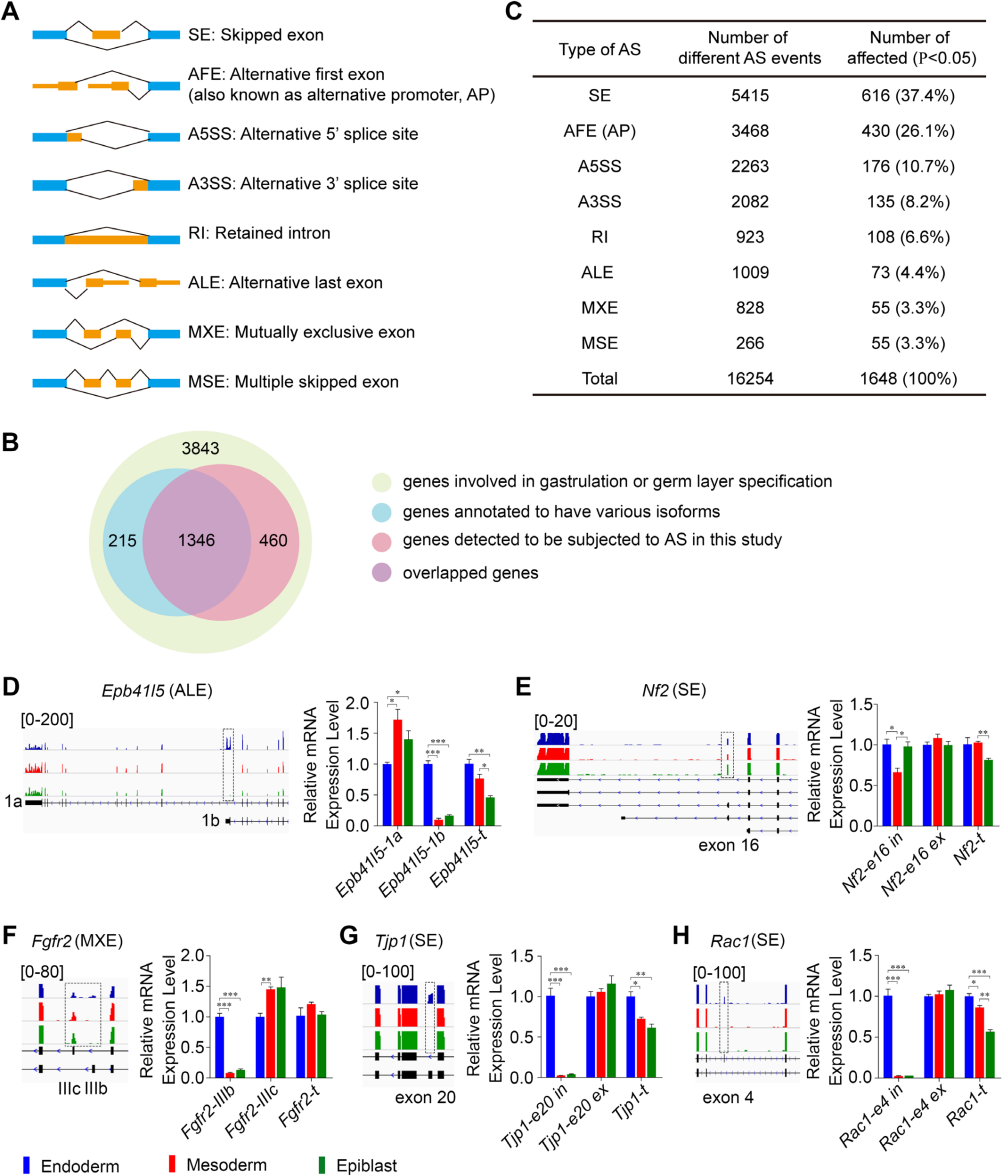
**Alternative splicing signature of the three germ layers.** (A) Diagrams of the eight types of AS analyzed in the study. Blue boxes, the constitutive exons; orange boxes, alternatively spliced exons/regions; solid lines, splice junctions. (B) Comparison of genes that are involved in gastrulation or germ layer specification and annotated to have different isoforms with those detected to be subjected to certain AS events using ASD. 1,346 out of 1,561 genes with annotated isoforms could be detected in our study. (C) The significantly different AS events in the three germ layers identified using ASD software (adjusted *P*-value<0.05). The number and percentage of the representative eight types of AS events are shown. (D-H) Validation of diverse AS events in genes that are functional at E7.5 using qPCR. In each case, the differential spliced exons detected by ASD and visualized using IGV are labeled by dotted boxes. The colored peaks represent the cover heights of the position (left panels). The AS events and the total expression level of each gene were analyzed using qPCR with isoform-specific primers or common primers (*P<0.05, **P<0.01, ***P<0.001) (right panels). Alternative last exons were labeled as 1a and 1b, respectively. The types of AS are shown in parentheses. ALE, alternative last exon; SE, skipped exon; MXE, mutually exclusive exon; e, exon; t, total; in, inclusion; ex, exclusion. Error bars represent s.e.m.; *n*=3.

There are four major types of AS events in mammals, including skipped exon, alternative 3′ splice site, alternative 5′ splice site, and retained intron. Skipped exon is the most common type, accounting for nearly 40% of all known AS events (Keren et al., 2010). Other less frequent AS events include mutually exclusive exon, multiple skipped exon, alternative first exon (also known as alternative promoter usage, AP usage) and alternative last exon (Keren et al., 2010) (Fig. 2A). To investigate the potential involvement of AS in mouse early embryogenesis, we used the Alternative Splicing Detector (ASD), also known as Comprehensive AS Hunting (CASH), a junction reads-based AS site construction Java program, to identify and compare the above eight types of AS events among the three germ layers (Wu et al., 2017; Zhou et al., 2014).

A total of 16,254 splicing events were detected from different germ layers using ASD. We first evaluated whether ASD was able to detect known AS events of genes expressed in E7.5 mouse embryos. Based on the Genes and Markers Query Form of MGI (Mouse Genome Informatics), a list of 3,843 genes that are potentially involved in ‘gastrulation’ or ‘germ layer specification’ biological processes were extracted (Fig. 2B; Table S1), and should be expressed at E7.5. Among the 3,843 genes, 1,561 genes were annotated to be regulated by AS and have different isoforms (Fig. 2B; Table S1). In our study, 1,806 out of the 3,843 genes were detected to be subjected to different AS events that covered 86.2% (1,346/1,561) of the 1,561 annotated alternatively spliced genes, including many experimentally validated events, such as the alternative promoter usage of *Otx2* (Acampora et al., 2009; Fossat et al., 2005), skipped exon and alternative 5′ splice site of *Fgfr1* (Partanen et al., 1998; Xu et al., 1999), skipped exon of *Smad2* (Dunn et al., 2005), alternative 5′ splice site, alternative 3′ splice site and skipped exon of *Fgf8* (Macarthur et al., 1995), mutually exclusive exon of *Fgfr2* (Orr-Urtreger et al., 1993), and skipped exon of *Dab2* etc (Maurer and Cooper, 2005) (Fig. 2B; Table S1). Thus, the method we used was sensitive enough to detect known and potential novel AS events.

With an adjusted *P*-value cutoff of <0.05, 10.1% AS events (1,648/16,254) were found to be significantly different between different germ layers (Fig. 2C; Table S1). These AS events include 616 (37.4%) skipped exon, 430 (26.1%) AP usage, 176 (10.7%) alternative 5′ splice site, 135 (8.2%) alternative 3′ splice site, 108 (6.6%) retained intron, 73 (4.4%) alternative last exon, 55 (3.3%) mutually exclusive exon and 55 (3.3%) multiple skipped exon (Fig. 2C). Several representative differential alternatively spliced events of genes known to be functional at gastrulation can be successfully validated in our study, including sipped exon of *Tjp1*, *Rac1* and *Nf2*, alternative last exon of *Epb41l5* and mutually exclusive exon of *Fgfr2* (Fig. 2D-H) (Arman et al., 1998; Hirano et al., 2008; Katsuno et al., 2008; McClatchey et al., 1997; Sugihara et al., 1998). In summary, diverse types of AS occur and are differentially present between E7.5 mouse primary germ layers, indicating the prevalence and potential implications of AS in regulation of development at this stage.

### Alternatively spliced genes are extensively involved in important developmental processes

**Fig. 3.**
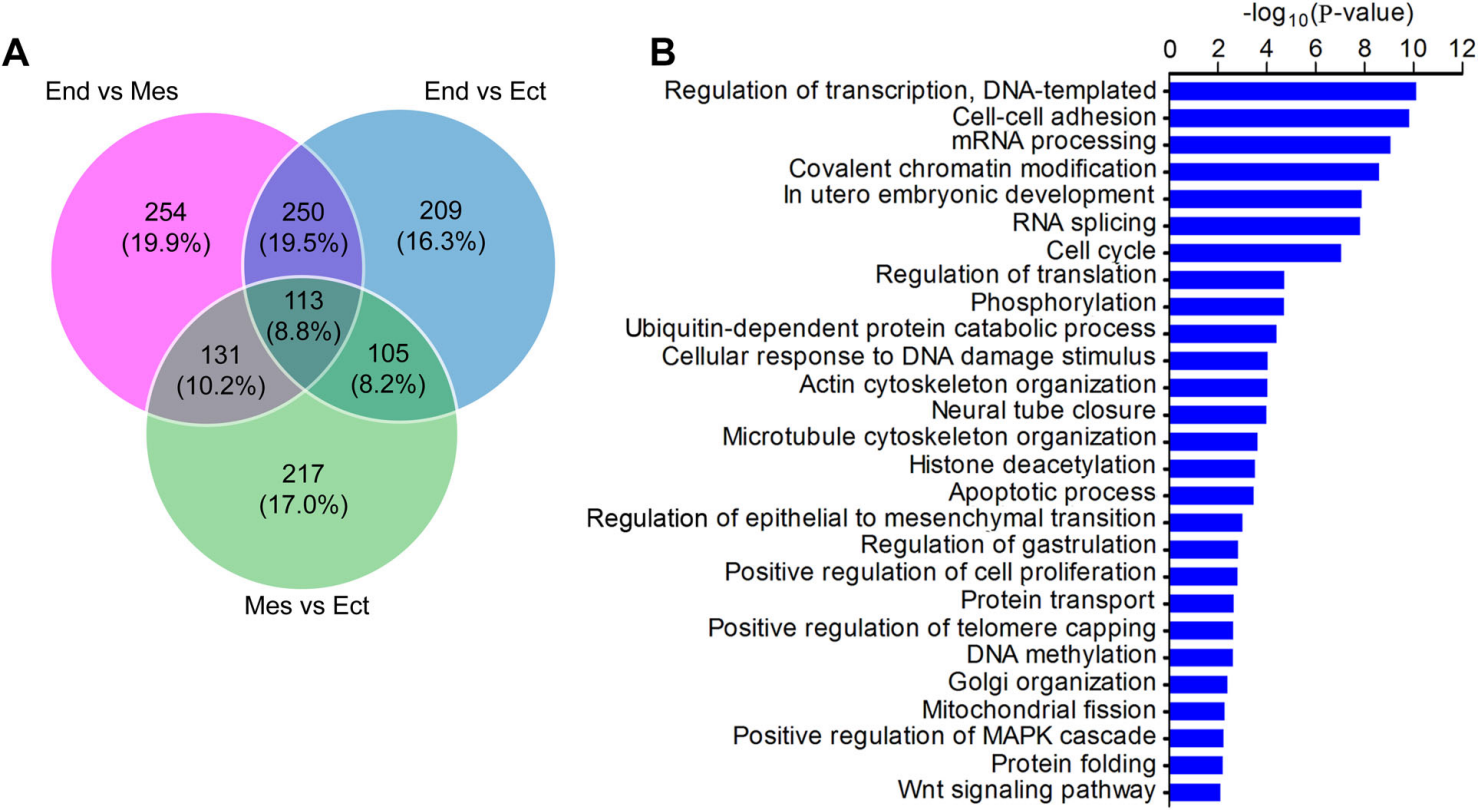
**Alternatively spliced genes are extensively involved in key developmental processes.** (A) Analysis of the genes with significantly different AS events (adjusted *P*-value<0.05) shared by the three germ layers using Venn diagram. (B) GO term enrichment analysis of the 1,279 genes generating the 1648 AS events (*P*<0.01). End, endoderm; Mes, mesoderm; Epi, epiblast.

The 1,648 significantly differential AS events were generated by 1,279 genes (Fig. 3A; Table S1). To determine whether these genes with alternative isoforms play a role in mammalian early development, we used DAVID for GO analysis (Huang et al., 2009). The result demonstrated that the 1,279 genes participated in numerous events, including regulation of transcription and translation, epigenetic modification (chromatin modification, DNA methylation, histone deacetylation), protein metabolism, organelle organization and Wnt cell signaling etc. (*P*<0.01) (Fig. 3B). In addition, developmental processes, such as *in utero* embryonic development, neural tube closure, epithelial-mesenchymal transition and gastrulation were also overrepresented in the GO terms (Fig. 3B), consistent with the embryogenic development at E7.5 stage. Therefore, these results suggest that AS machinery might be widely used and relevant to certain biological processes during specification of the germ layers and subsequent organogenesis.

### Splicing factors are differentially expressed and alternatively spliced across the germ layers

**Fig. 4.**
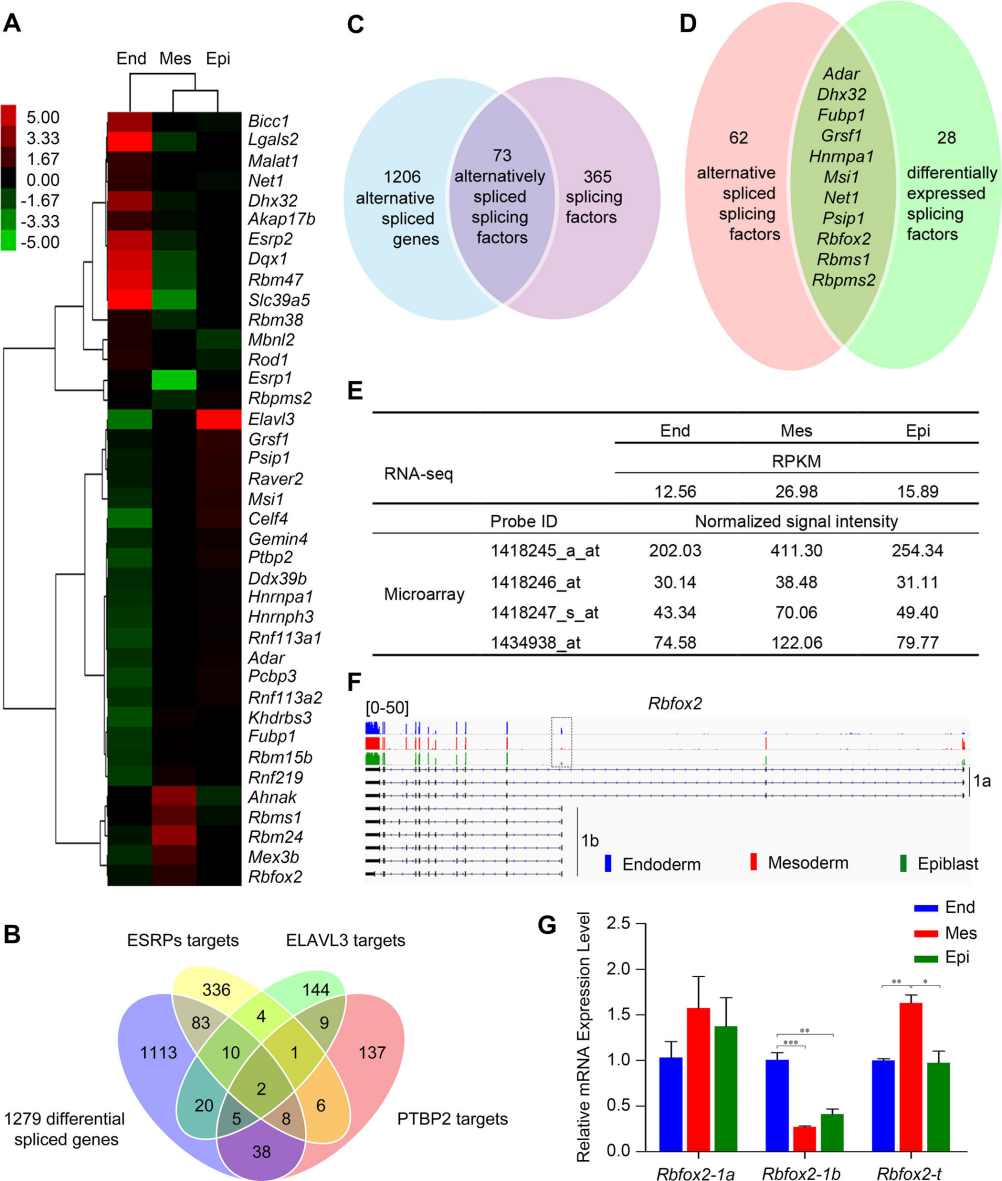
**Splicing factors are differentially expressed and alternatively spliced across the germ layers.** (A) The heat map of 39 significantly differentially expressed RNA splicing factors in the germ layers (at least one RPKM>5, Fold Change>2, FDR<0.001). (B) Venn diagram showing the targets of ESPRs (ESRP1 and ESRP2), ELAVL3 and PTBP2 in the 1,279 differential alternatively spliced genes. (C) Venn diagram showing the 73 alternatively spliced splicing factors. (D) Venn diagram showing the 11 differentially expressed and simultaneously alternatively spliced splicing factors. The name of the 11 splicing factors was shown. (E) The RPKM value and normalized signal intensity of *Rbfox2* in the RNA-seq and Microarray data, respectively. Four probes were used in the Microarray analysis to detect *Rbfox2*. (F) The differential AS events of *Rbfox2* between the three germ layers visualized using IGV software with RNA-seq mapped reads. The differential spliced exon detected by ASD is labeled by dotted boxes. The alternative first exons due to selective use of distal and proximal promoters are labeled as 1a and 1b, respectively. The colored peaks in each case represent the cover heights of the position. (G) Validation of the AS events and the total expression level of *Rbfox2* using qPCR with isoform-specific primers or common primers (*P<0.05, **P<0.01, ***P<0.001). End, endoderm; Mes, mesoderm; Epi, epiblast. Error bars represent s.e.m.; *n*=3. See also Fig. S2.

Splicing factors are often involved in tissue- or cell-specific AS patterns. We reasoned that the variant presence of AS events across the three germ layers may lie in the differential expression of splicing factors. To test this idea, we examined the expression of a list of 438 known or putative splicing factors that were previously used for analysis (Goldstein et al., 2017; Han et al., 2013), or fell into ‘RNA splicing’- and/or ‘spliceosome’-associated GO terms in our study (Table S1). RNA-seq expression profiling revealed that 39 out of the 438 splicing factors exhibited significant differential expression among different germ layers (at least one RPKM>5, Fold Change>2, FDR<0.001) (Fig. 4A). In particular, these genes include many well documented cell- or tissue-specific alternative splicing factors like *Esrp1*, *Esrp2*, *Khdrbs3* and *Celf4* (Chen and Manley, 2009). *Esrp1* and *Esrp2* have been reported to be involved in epithelial-specific RNA splicing and related with the splicing pattern change during the epithelial-to-mesenchymal transition (Warzecha et al., 2009a). We found a significant downregulation of these two genes, especially *Esrp1* in mesoderm (Fig. 4A), which might make way for establishment of mesodermal AS patterns and help to define mesodermal cell characteristics during the epithelial-to-mesenchymal transition of gastrulation. Additionally, when comparing the 1,279 differential alternatively spliced genes with previously reported data, we found that 103, 35 and 53 genes were overlapped with the potential targets of ESRPs (ESRP1 and ESRP2) (450 potential targets) (Bebee et al., 2015; Warzecha et al., 2009b), ELAVL3 (195 potential targets) (Ince-Dunn et al., 2012) and PTBP2 (206 potential targets) (Boutz et al., 2007), respectively (Fig. 4B; Table S1). Thus, apart from constitutive splicing, variant presence of splicing factors might account for the different AS efficiencies and patterns across the three samples and therefore the distinct developmental consequences.

When performing GO analysis of the 1,279 alternatively spliced genes, we observed a highly significant enrichment of genes associated with RNA processing and/or splicing (Fig. 3B). Consistently, Venn diagram analysis showed that among the 1,279 genes that generate differential AS events in different germ layers, 73 genes encode splicing factors (Fig. 4C). These data indicate that AS is superimposed on the functions of RNA splicing factors themselves, which is consistent with previous reports that many splicing factors are affected by alternative splicing and may be involved in self-regulatory feedback loops (Sureau et al., 2001; Valacca et al., 2010). Moreover, if taking gene expression and alternative splicing together for consideration, 11 splicing factors were differentially expressed and simultaneously exhibited significantly differential AS patterns across different germ layers (Fig. 4D, Table 1). One of the candidates is RBFOX2 [RNA binding protein, fox-1 homolog (*Caenorhabditis* *elegans*) 2], also known as FXH or RBM9, and plays essential roles in alternative exon splicing in mammalian cells (Nakahata and Kawamoto, 2005). According to our RNA-seq and Microarray data, the expression level of *Rbfox2* was higher in mesoderm compared to that in endoderm and epiblast (Fig. 4E). Meanwhile, AS analysis showed that the inclusion of an alternative first exon due to use of the proximal promoter (1b) was much higher in endoderm than that in mesoderm or epiblast (Fig. 4F, Table 1), while *Rbfox2* isoforms in the mesoderm and epiblast tended to use the distal promoter (1a) (Fig. 4F). The total expression level of *Rbfox2* and differential use of APs in different germ layers were confirmed using qPCR (Fig. 4G). It was reported that *Rbfox2* underwent tissue-specific alternative splicing and generated tissue-specific isoforms that mediate brain- and muscle-specific AS events and thus help to define their specificity (Nakahata and Kawamoto, 2005). Likewise, it is possible that differential expression of *Rbfox2* and differential use of APs in different germ layers contribute to establishment of germ layer-specific AS events and commitment of cell fate.

**Table 1.**
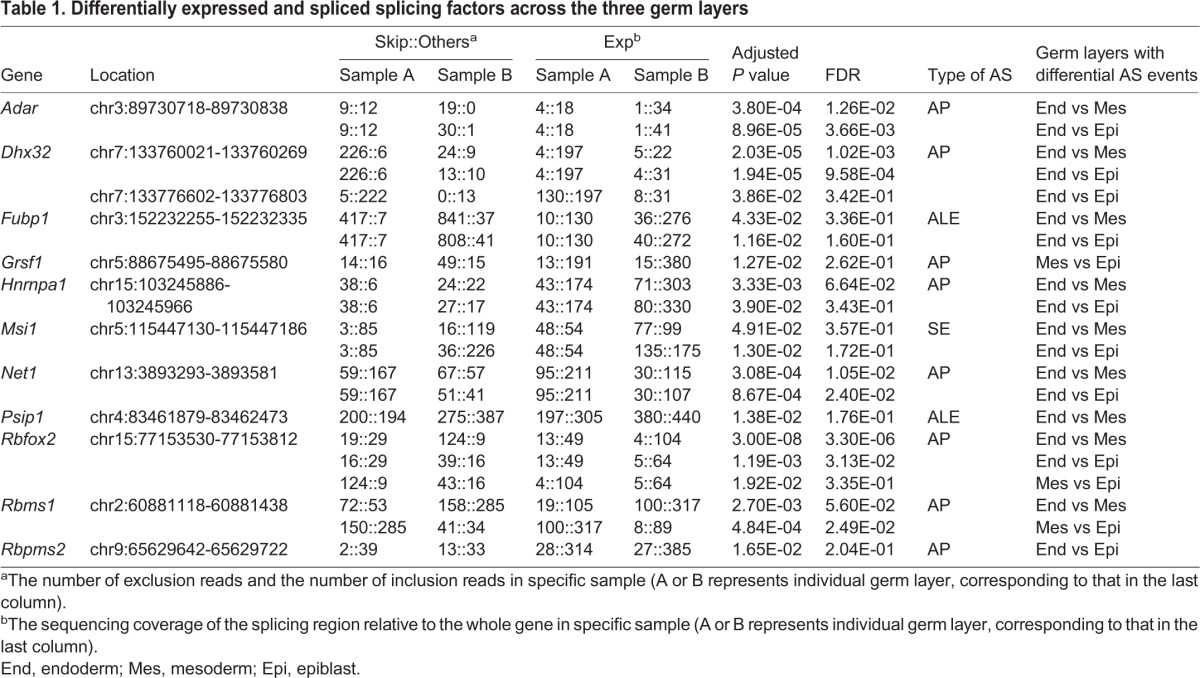
**Differentially expressed and spliced splicing factors across the three germ layers**

Altogether, many splicing factors are differentially expressed and have distinct level or types of alternative isoforms in the three germ layers, which might help to establish specific AS events and thus define tissue specificity of each germ layer.

### Alternative promoter usage is frequent in the three germ layers of E7.5 mouse embryos

As aforementioned, skipped exon, alternative 3′ splice site, alternative 5′ splice site, and retained intron are the most common AS types in mammals, with skipped exon (i.e. cassette) the most prevalent one (Sammeth et al., 2008). In concordance with this, the majority of the 1,648 differentially presented AS events (616/1,648) in E7.5 mouse germ layers were exon skipping (Fig. 2B). Strikingly, however, secondary to exon skipping, more than one quarter of events (430/1,648) belonged to AP usage, which is rarely documented in other processes. Of interest, among the 11 differentially expressed and simultaneously alternatively spliced splicing factors mentioned above, we found that AP usage was the most common AS event (8/11) (Table 1). These data indicate that AP usage is frequent and might be a prevalent mechanism of gene expression regulation in spatially controlling mouse early embryonic development.

**Fig. 5.**
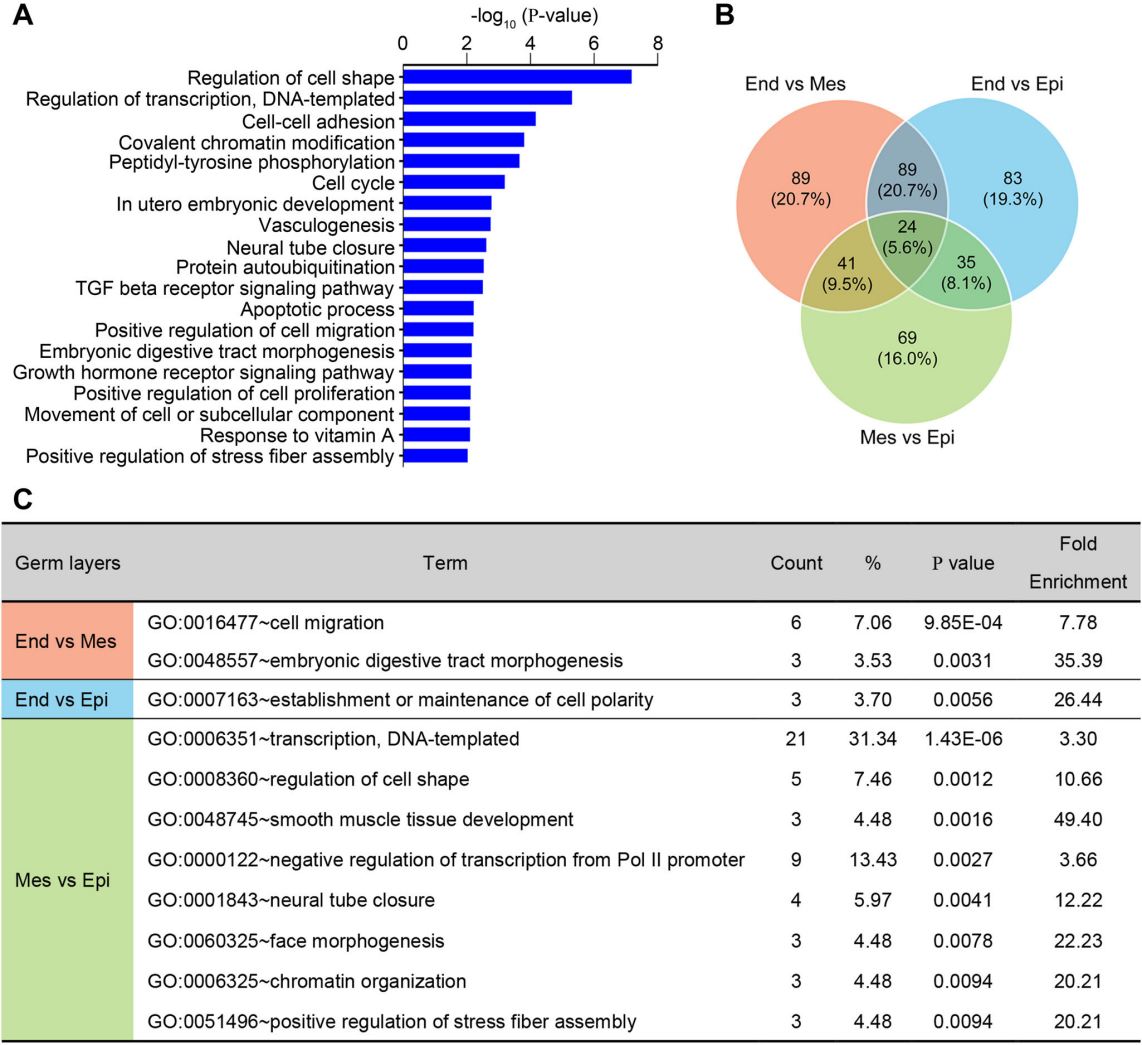
**AP usage is a prevalent machinery in controlling gene expression in post-gastrulation embryos.** (A) GO term enrichment analysis of the 383 genes generating the 430 AP events (*P*<0.01). (B) Venn diagram of the significantly different AP usage events in the three germ layers (adjusted *P*-value<0.05). (C) Functional enrichment of GO terms for genes showing differential AP events between endoderm and mesoderm (End versus Mes), endoderm and epiblast (End versus Epi), and mesoderm and epiblast (Mes versus Epi), respectively.

AP usage is postulated to be frequently observed in developmentally spatio-temporal manners but has not been well studied (Forrest et al., 2014). The 430 AP events in our study were generated by 383 genes (Table S1), GO analysis of which revealed that they participated in numerous development-related biological processes, including regulation of cell shape, transcription, chromatin modification, protein metabolism, TGFβ cell signaling, proliferation and apoptosis etc. (*P*<0.01) (Fig. 5A). When AP usage events were compared in pairs of the three germ layers, 89, 83 and 69 events were specifically significantly different between endoderm versus mesoderm, endoderm versus epiblast, and mesoderm versus epiblast, respectively (Fig. 5B). Genes generating these events were involved in processes that matched well with germ layer-specific developmental events. For example, the 89 different AP events between endoderm and mesoderm were generated by 85 genes. GO analysis revealed that these genes were enriched in ‘cell migration’ and ‘embryonic digestive tract morphogenesis’, which echoed the epithelial-mesenchymal transition of mesoderm cells and the commitment of endoderm, respectively (Fig. 5C). Likewise, genes generating the 69 AP events between mesoderm and epiblast were overrepresented in muscle and neuro development (Fig. 5C). Thus, the frequent use of AP suggests that promoter choice might be an important regulatory mechanism for mouse early embryonic development.

### Validation of the AP usage events in E7.5 mouse embryonic germ layers

**Fig. 6.**
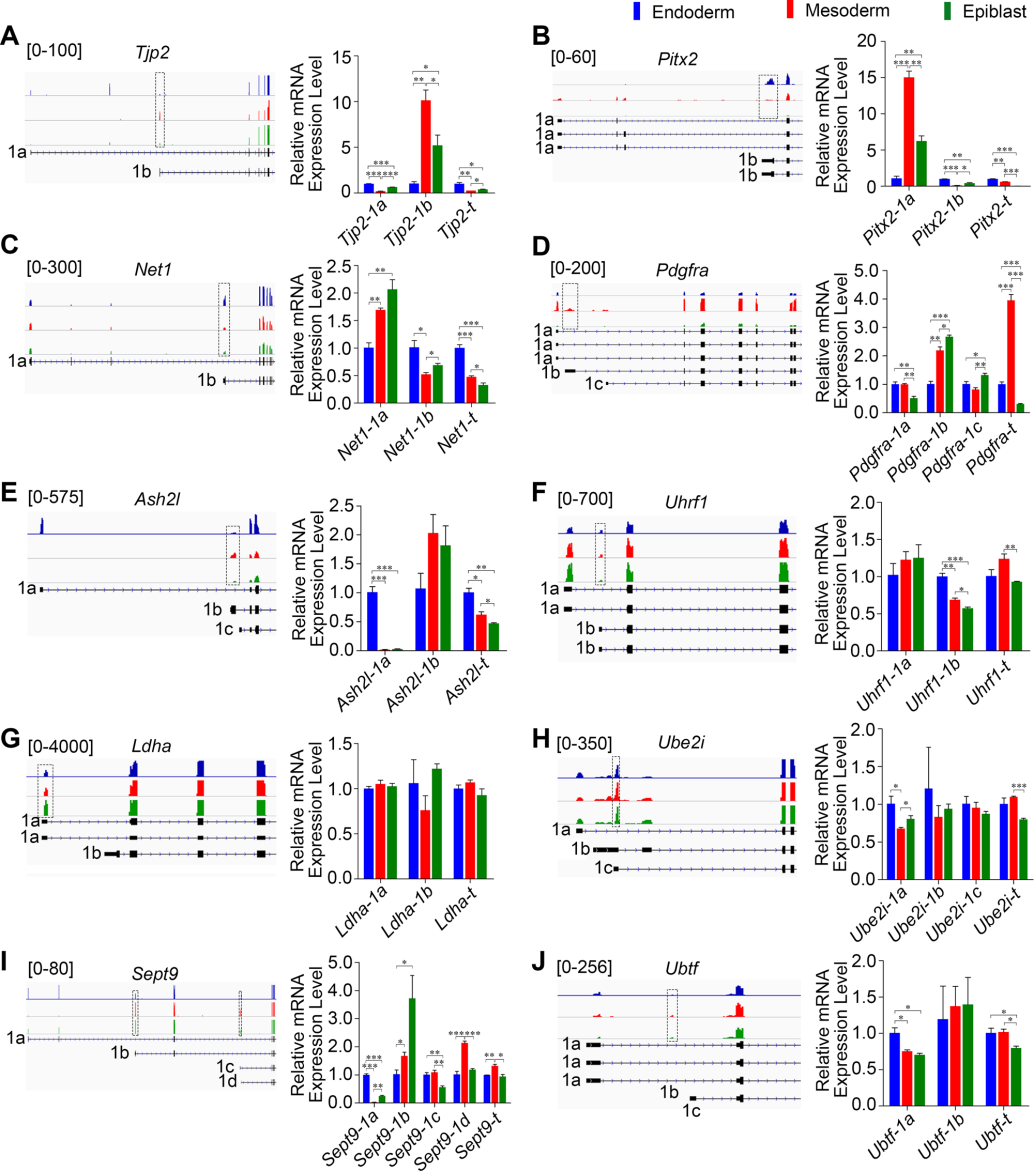
**Validation of the AP usage events in E7.5 mouse embryonic germ layers using qPCR.** (A-J) Genes with significantly different AP events across the three germ layers identified by RNA-seq data analysis using IGV software. In each case, the positions of the predicted differential first exons generated by APs are labeled by dotted boxes. The colored peaks represent the cover heights of the position (left panels). The AP events and total expression level of each gene were analyzed using qPCR with exon-specific primers and common primers, respectively (*P<0.05, **P<0.01, ***P<0.001) (right panels). Alternative first exons in each gene were labeled as 1a, 1b, 1c and 1d, respectively. No coverage of *Ash2l-1c* and *Ubtf-1c* was detected by IGV. Error bars represent s.e.m.; *n*=3.

Among the 430 AP events, we found that many events were generated by well characterized regulators of gastrulation or beyond, such as *Tjp2* (Xu et al., 2008), *Pitx2* (Kitamura et al., 1999; Lin et al., 1999), *Net1* (Nakaya et al., 2008) and *Pdgfra* (Funa and Sasahara, 2014). The isoforms of these genes generated by APs were predicted to be distinctly enriched in the three germ layers and were visualized by IGV (Integrative Genomics Viewer) tool (Fig. 6A-D, left panels). The computational results were then analyzed using qPCR (Fig. 6A-D, right panels). To simplify the results, the ASD software was designed to locate the relatively inner exon when addressing AP and ALE events (Wu et al., 2017; Zhou et al., 2014). Thus, besides the different positions detected by ASD, we examined all the possible isoforms generated by APs with exon-specific primers, except for those with no detectable coverage. In good concordance with what was observed using ASD and IGV (Table 2), the qPCR result showed that all cases could be well validated (Fig. 6A-D).

**Table 2.**
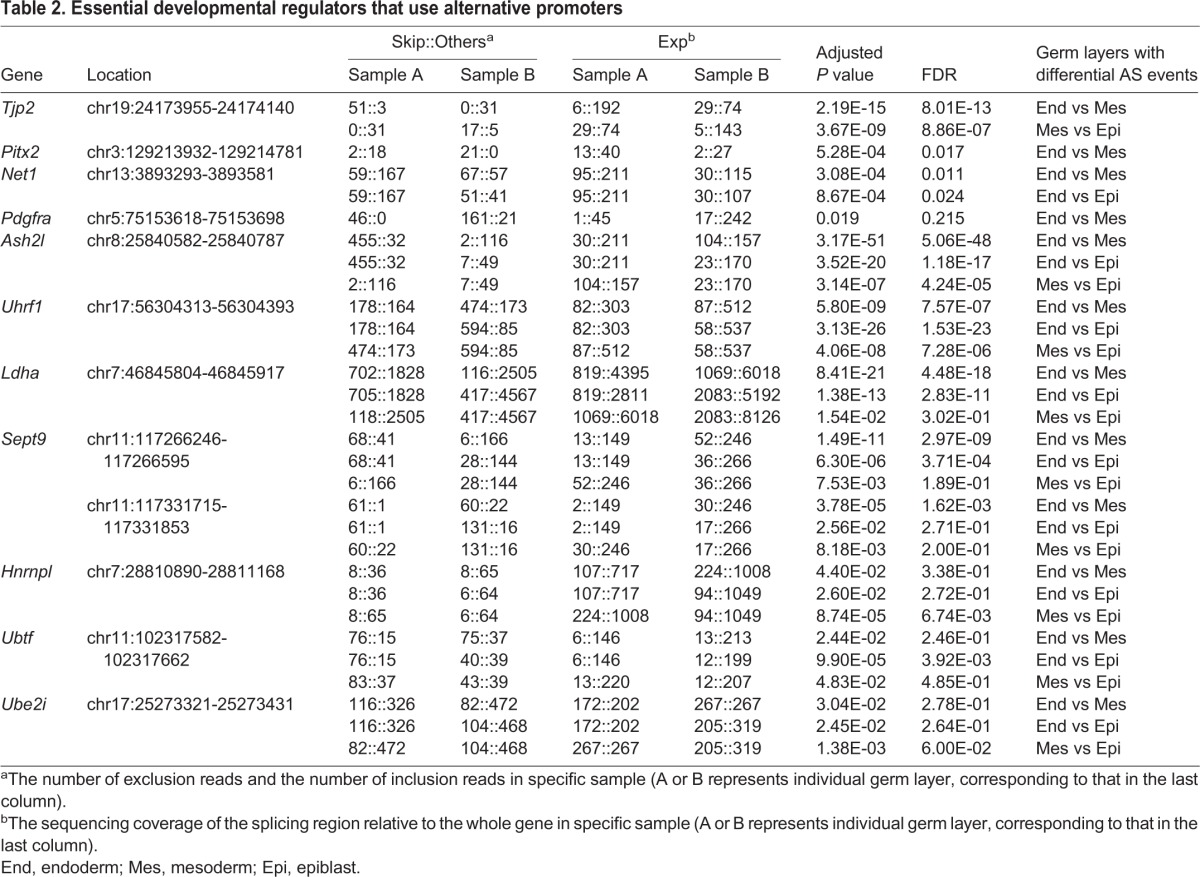
**Essential developmental regulators that use alternative promoters**

To further confirm the frequent AP events, we focused on additional 24 AP events, which involved 23 genes that were shared by all the three germ layers (Fig. 5B; Table S1). To narrow the genes to a range suitable for AS events confirmation and function study, we analyzed those that have annotated isoforms and potentially play important roles in mouse early embryogenesis. Among the 23 genes, *Ash2l*, *Uhrf1*, *Ldha*, *Sept9*, *Hnrnpl*, *Ubtf* and *Ube2i* are required for cell survival, as analyzed using Batch Query tool of Mouse Genome Informatics (MGI) (Table S1). Except for *Hnrnpl*, which has only one annotated transcript, the isoforms of the other six genes generated by APs were predicted to be expressed in germ layer-specific manners (Fig. 6E-J). The qPCR result showed that at least one isoform of *Ash2l*, *Uhrf1*, *Ube2i*, *Sept9* and *Ubtf* was significantly differentially expressed in the three germ layers (Fig. 6E-J).

Among these genes, *Tjp2*, *Pitx2*, *Net1*, *Ash2l*, *Uhrf1* and *Sept9* have been previously reported to generate different isoforms driven by APs (Chlenski et al., 2000; Connolly et al., 2011; Hopfner et al., 2001; Qin et al., 2005; Sørensen et al., 2002; Waite et al., 2013; Wang et al., 2001). Importantly, the APs directed isoforms of *Net1*, *Pitx2* or *Sept9* exhibited different cellular or subcellular localization, and functioned in isoform-specific manners (Carr et al., 2013; Connolly et al., 2011; Dutertre et al., 2010; Papadimitriou et al., 2012; Sørensen et al., 2002; Waite et al., 2013). For example, NET1A, the shorter protein isoform resulting from usage of the proximal promoter (1b) of *Net1*, is specifically required for actin cytoskeletal reorganization, myosin light chain phosphorylation, and focal adhesion maturation, controlling cell spreading or motility (Carr et al., 2013; Papadimitriou et al., 2012). The longer isoform generated from the distal promoter (1a), however, is more important for cell proliferation (Dutertre et al., 2010). Likewise, the isoforms of *Sept9*, a member of the *Septin* gene family, exhibit distinct expression patterns in adult mouse tissues and in tumor cells, and exert different influence on breast cancer progression (Connolly et al., 2011; Sørensen et al., 2002; Verdier-Pinard et al., 2017). While human *Sept9_v1* (homologous to *Sept9-1a*) promotes breast cancer cell migration, *Sept9_v2* (homologous to *Sept9-1b*) seems to have little supportive or even antagonistic effect on breast tumorigenesis (Connolly et al., 2011; Verdier-Pinard et al., 2017). Collectively, these data support the prevalent use of APs by many key development-related genes and hence the potential developmental implications of AP usage in mouse early embryogenesis.

## DISCUSSION

AS of pre-mRNAs and AP usage are important machineries in regulation of gene expression in numerous processes (Ayoubi and VanDeVen, 1996; Davuluri et al., 2008; Kalsotra and Cooper, 2011; Sebastian et al., 2013). However, their regulatory functions and patterns in mammalian early embryonic development are still elusive. In this study, we analyze the AS events with RNA-seq data from the three embryonic germ layers of E7.5 mouse embryos and provide a landscape of AS patterns, and their potential regulation of development in gastrulating and post-gastrulation mouse embryos. We find that AS is widely used and may be involved in regulation of multiple processes in mouse early embryogenesis. Importantly, we discover for the first time that AP usage, which is not commonly observed in other processes, is frequent in the three germ layers, accounting for more than one quarter of the total significantly changed AS events in the three germ layers. These AP events are generated by genes that are involved in germ layer-specific cell fate commitment and include many key developmental regulators. Current paradigm highlights the importance of gene expression changes in control of development. As an important complement, our study underlines the biological implications of AS, especially AP usage in directing mammalian early development, which deserves to be carefully examined and confirmed in future developmental studies. Moreover, our study provides a useful data source for the analysis of gene or AS regulation of early mouse development.

Many studies have described and experimentally proved the requirement of correct spatial and temporal expression of alternative isoforms of individual genes in development and diseases (Baralle and Giudice, 2017). In our study, we find many splicing factors exhibit differential expression pattern across E7.5 mouse primary germ layers, at both whole transcript level and isoform level. Alternative splicing is often under the control of specific splicing factor, which may affect many AS events and have numerous target genes. Changes in their expression level or different presence of alternative isoforms can therefore lead to profound consequences. We did find many targets of the differentially expressed splicing factor ESRP1, ESRP2, ELAVL3 and PTBP2 in our differential alternatively spliced gene list (Fig. 4B; Table S1). In addition, apart from RBFOX2, a well-known splicing factor that we exemplified above (Fig. 4E-G), two other splicing factors, Adenosine deaminase, RNA specific (ADAR) and G-rich sequence factor 1 (GRSF1) in our list (Table 1; Fig. S2), were recently reported to be differentially expressed and spliced in humans, and play isoform-specific roles in different pathways or cellular compartment (Jourdain et al., 2013; Pestal et al., 2015). Thus, we postulate that differences at the global expression and isoform levels of splicing factors may underlie the germ layer-specific AS pattern and thus determine the following developmental process. However, direct experimental evidences are needed to support this notion.

Cell lineage specification during gastrulation is a highly dynamic and complex process involving drastic cell proliferation, migration and differentiation (Nowotschin and Hadjantonakis, 2010; Takaoka and Hamada, 2012; Tam and Behringer, 1997), which requires precise gene regulation. Usage of APs generally makes it feasible and flexible for transcription factors and epigenetic regulator to gain access to the regulatory elements or regions of a single gene locus and modulate the expression of the required mRNA variants in the right tissues at the right time (Davuluri et al., 2008). For example, it has been experimentally evidenced that usage of APs facilitates the control of expression of the required *Runx1* isoform by DNA methylation during hematopoiesis (Webber et al., 2013). Although there is no direct experimental evidence at present, clues from ChIP-seq data that are publicly available show that a similar mechanism might be applicable to the regulation of isoforms expression in *Sept9*, a candidate in our study (http://genome.ucsc.edu/) (Creyghton et al., 2010; Heintzman et al., 2009; Kim et al., 2007; Rada-Iglesias et al., 2011; Visel et al., 2009; Wu et al., 2011). While the transcriptionally permissive H3K4me3 modification and RNA polymerase II are highly enriched in all the annotated promoters (1a-1d) of *Sept9* in mouse embryonic stem cells, they are only enriched in the proximal three promoters (1b-1d) in mouse embryonic fibroblasts, brain and spleen, and enriched in the distal two promoters (1a and 1b) in kidney and liver (Fig. S3). In heart, however, only the 1b promoter has higher peaks of H3K4me3 modification and RNA polymerase II (Fig. S3). These different histone modifications and transcription factors binding at the alternative promoters of *Sept9* might contribute to the specific expression patterns of its different isoform in different tissues or cell lines. Thus, the prevalence of AP usage observed in the three germ layers might be an adaptive machinery to guarantee precise gene expression and successful development. Actually, prevalent use of APs has also been observed in six major tissues comprising most mass of the mid-gestational mouse embryo (Werber et al., 2014), further supporting the potential importance of AP usage in early mouse development.

High-throughput sequencing has uncovered an increasing number of genes undergoing alternative splicing and containing more than one promoter in mammalian genomes (Davuluri et al., 2008). However, our knowledge about the biological implications of alternative splicing, especially the selective usage of promoters and how and on what occasions they are differentially regulated, are still very limited. Our study, for the first time, provides an overview of alternative splicing in primary embryonic germ layers of mouse gastrulation and highlights the potential importance of AP usage at this stage and beyond, expanding the understanding of regulatory mechanisms of mammalian early embryonic development. However, the difficulty of complete microdissection of germ layers and limitation of current sequencing and AS analysis methods might make it challenging to obtain a thorough and definite landscape of AS patterns during gastrulation. We expect that further disintegrating the germ layers into single cells followed by single-cell long-read sequencing would provide a clearer picture.

## MATERIALS AND METHODS

### Animals

Mice were maintained under specific-pathogen-free (SPF) conditions and all animal experiments were performed in compliance with the guidelines of the Animal Care and Use Committee of the Institute of Zoology, Chinese Academy of Sciences.

### Embryonic germ layer separation

Pregnant ICR female mice at 8 days post coitus (d.p.c.) were killed. E7.5 mouse embryos were dissected from the deciduas in HEPES-buffered DMEM supplied with 10% FBS. After Reichert's membrane was removed, the embryonic region was cut off using a glass scalpel. The embryonic region was then rinsed in Dulbecco's phosphate-buffered saline (DPBS) and incubated in pancreatic/trypsin enzyme solution (0.5% trypsin and 2.5% pancreatin in Ca^2+^/Mg^2+^-free Tyrode Ringer's saline, pH 7.6-7.7) at 4°C for 10 min. After the treatment, the embryonic region was transferred into HEPES-buffered DMEM supplied with 10% FBS. The germ layers were separated carefully with a Pasteur pipette and glass needles.

### Microarray and data analysis

Total RNA from each germ layer was extracted using RNeasy Micro Kit (Qiagen, 74004) according to the manufacturer's instructions and treated with DNase I to remove residual genomic DNA. After verifying the RNA integrity and amount with an Agilent Bioanalyzer 2100 instrument, gene expression profiling was performed using an Affymetrix Mouse Genome 430 2.0 Array (CapitalBio, Beijing, China) with a starting RNA amount of 100 ng. The raw hybridization data were analyzed using Affymetrix GeneChip Command Console Software (AGCC). The Robust Multiarray Average (RMA) method was used for background correction and data normalization, and the affy suite of the bioconductor package (http://www.bioconductor.org) was used to calculate expression values. Significance Analysis of Microarrays (SAM) was used to identify genes that are differentially expressed.

### RNA sequencing and data analysis

Total RNA from each germ layer sample was prepared using RNeasy Micro Kit (Qiagen, 74004) according to the manufacturer's instruction and treated with DNase I to remove residual genomic DNA. After quality control using Agilent 2500, cDNA libraries for paired-end sequencing were prepared using BGI Kit (Beijing Genomics Institute, Beijing, China) and sequencing was performed on an Illumina HiSeq™ 2000 system. For each sample, about 4G clean data were generated after removing the adapter sequences and low-quality reads. Differentially expressed genes were analyzed as previously reported with a *P*-value, and a false discovery rate (FDR) to correct for the *P*-value (Audic and Claverie, 1997; Benjamini and Hochberg, 1995; Benjamini and Yekutieli, 2001).

### Alternative splicing analysis

AS patterns were analyzed using ASD software as previously described (Wu et al., 2017; Zhou et al., 2014). Briefly, the clean reads were mapped to the mouse genome (Mouse GRCm38/mm10) by TopHat. The generated bam files that were sorted and indexed were imported and analyzed using the ASD software. Eight modes of AS events for the three germ layers were obtained with an adjusted *P*-value and false discovery rate (FDR). The Integrative Genomics Viewer (IGV) tool was used for efficient and flexible visualization of specific AS event. DAVID was employed to conduct GO term enrichment analysis.

### RNA extraction, reverse transcription and quantitative PCR

Total RNA was extracted using RNeasy Micro Kit (Qiagen, 74004) according to the manufacture's instruction. After treatment with DNase I to remove residual genomic DNA, 500 ng total RNA was used as template for 10 μl reverse transcription reaction using PrimeScript™ RT Reagent Kit (TaKaRa, Dalian, China, RR037A). Quantitative PCR was performed using EvaGreen 2×qPCR MasterMix (Applied Biological Materials, Vancouver, Canada, MasterMix-S) on LightCycler 480 instrument (Roche, Basel, Switzerland). Relative gene expression was analyzed based on 2^−ΔΔCt^ method. When analyzing the expression level of a specific isoform, the transcripts detected using a common pair of primers for all isoforms was used as internal reference, while the total level of individual gene was normalized to the expression level of *Gapdh*. All primers are listed in Table S3.

### Statistical analysis

Quantitative analyses were performed with at least three independent biological samples using GraphPad Prism software and expressed as mean±s.e.m. *P*-values of comparisons between two groups were calculated using Student's *t*-test with significance levels of *P<0.05, **P<0.01, ***P<0.001.

## Supplementary Material

Supplementary information
