# Supplementary material for: Whole-transcriptome splicing profiling of E7.5 mouse primary germ layers reveals frequent alternative promoter usage during mouse early embryogenesis
Source: Biol Open. 2018 Mar 15;7(3):bio032508. doi: 10.1242/bio.032508 (PMC5898269; doi:10.1242/bio.032508)
Supplement: Supplementary information [file biolopen-7-032508-s1.pdf]

SUPPLEMENTAL FIGURES

Supplementary Figure S1

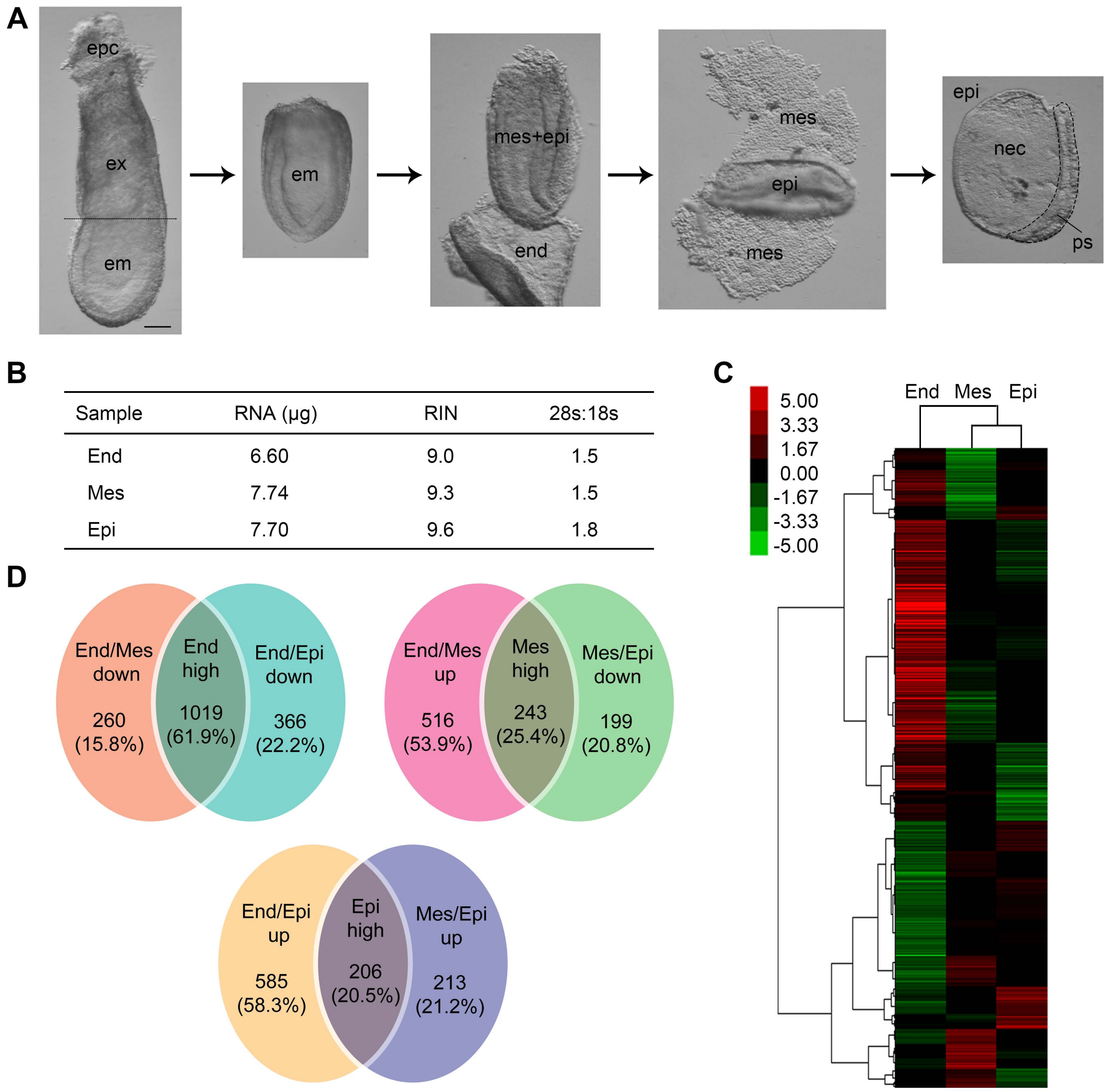

Sample	RNA (µg)	RIN	28s:18s
End	6.60	9.0	1.5
Mes	7.74	9.3	1.5
Epi	7.70	9.6	1.8

Biology Open • Supplementary information

Supplementary Figure S1. Transcriptome profiling of the three embryonic germ layers of E7.5 mouse embryos. Related to Figure 1.

(A) Schematic overview of the dissection of the three germ layers. The embryonic region was first separated from the extra-embryonic region. Then the embryonic endoderm, mesoderm and epiblast (neuroectoderm and primitive streak) were dissected carefully in sequence with mouth pipette and glass needles. epc, ectoplacental cone; ex, extra-embryonic region; em, embryonic region; end, endoderm; mes, mesoderm; epi, epiblast; nec, neuroectoderm; ps, primitive streak. The primitive streak was encircled with dotted line. Scale bar, 100 μm. (B) The amount and quality of RNAs of each germ layer. RIN, RNA integrity number. (C) The heat map of 2880 genes differentially expressed in the three germ layers of E7.5 mouse embryos. (D) Venn diagram showing genes highly expressed in each germ layer. Genes down-regulated in mesoderm when comparing endoderm and mesoderm (End/Mes down), and simultaneously down-regulated in epiblast when comparing endoderm and epiblast (End/Epi down) are regarded as with high expression level in endoderm (End high); genes up-regulated in mesoderm when comparing endoderm and mesoderm (End/Mes up), and simultaneously down-regulated in epiblast when comparing mesoderm and epiblast (Mes/Epi down) are regarded highly expressed in mesoderm (Mes high); genes up-regulated in epiblast when comparing endoderm and epiblast (End/Epi up), and simultaneously up-regulated in epiblast when comparing mesoderm and epiblast (Mes/Epi up) are regarded as with highly expressed in epiblast (Epi high).

Biology Open • Supplementary information

Supplementary Figure S2

A

		End	Mes	Epi
RNA-seq		RPKM		
		4.67	8.95	10.53
	Probe ID	Normalized signal		
Microarray	1425405_a_at	68.35	47.39	86.06
	1434268_at	184.05	245.77	269.20
	1439276_at	17.19	35.63	30.49

C

		End	Mes	Epi
RNA-seq		RPKM		
		41.52	52.48	99.88
	Probe ID	Normalized signal intensity		
Microarray	1437838_x_at	1654.49	2878.37	3576.02
	1433457_s_at	677.72	914.46	1432.19
	1433824_x_at	1150.77	1555.66	2106.33

B

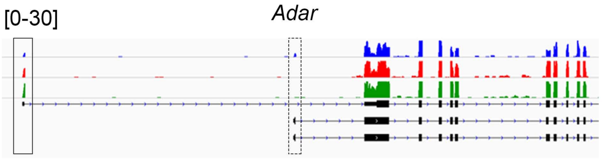

D

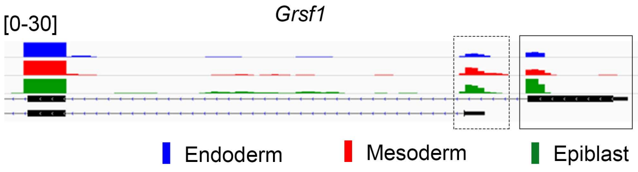

Supplementary Figure S2. ADAR and GRSF1 are differentially expressed and alternatively spliced across the germ layers. Related to Figure 4.

(A) The RPKM value and normalized signal intensity of *Adar* in the RNA-seq and Microarray data, respectively. Three probes were used in the Microarray analysis to detect *Adar*. End, endoderm; Mes, mesoderm; Epi, epiblast. (B) The differential AS events of *Adar* between the three germ layers visualized using IGV software with RNA-seq mapped reads. The positions of the proximal and distal promoters are labeled by dotted and solid boxes, respectively. The colored peaks in each case represent the cover heights of the position. (C and D) Same as A and B, except the gene is *Grsf1*.

Biology Open • Supplementary information

Supplementary Figure S3

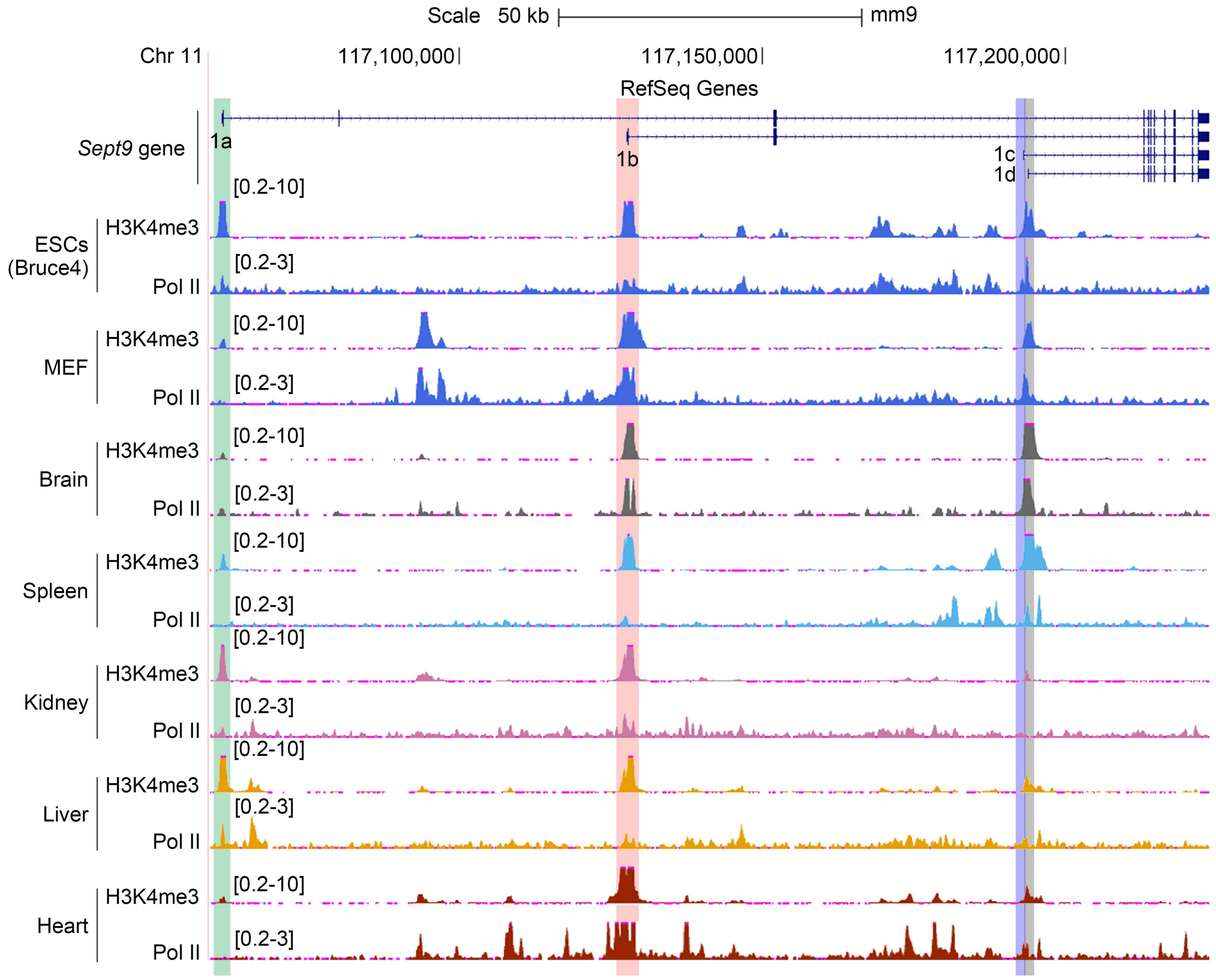

Supplementary Figure S3. Differential modification of H3K4me3 and binding of RNA polymerase II (Pol II) at the alternative promoters of *Sept9* in different cell lines and tissues. Data are extracted from UCSC (http://genome.ucsc.edu/). The regions of promoters are highlighted in different colors.

Supplementary Table S1: Gene list

Click here to Download Table S1

Supplementary Table S2. RNA-seq data mapping results.

Sample	Endoderm	Mesoderm	Ectoderm
Clean reads	49744660 (100%)	49753030 (100%)	51036608 (100%)
Total mapped reads	41988506 (84.4%)	42140982 (84.7%)	43225419 (84.7%)
Unique mapped	37188205 (74.8%)	36384199 (73.1%)	36965503 (72.4%)
Multiple mapped	4800301 (9.7%)	5756783 (11.6%)	6259916 (12.3%)
Total unmapped reads	7756154 (15.6%)	7612048 (15.3%)	7811189 (15.3%)

Biology Open • Supplementary information

Supplementary Table S3. Primers used for quantitative real-time PCR

Genes	Direction	Sequence (5' to 3')
Ash2l-1a	F	ATGAAGGAGGAGGCCAGGAC
	R	CCGCCTGGGTATCCATCAC
Ash2l-1b	F	GAGGACCGAGAGACAGAGCC
	R	AGTTTGCATCCCCACTTTCG
Ash2l-t	F	TGGGCGGGAAAGCCTATT
	R	CACGGTCAGCCGGTCATC
Ldha-1a	F	GGCCACGCTGCTTCTCCT
	R	TTGTTCTGGGGAGCCTGCT
Ldha-1b	F	GCGGCTACACGTACACGGA
	R	TTGTTCTGGGGAGCCTGCT
Ldha-t	F	TGTTGGGGTTGGTGCTGTT
	R	TCATCTCGCCCTTGAGTTTG
Sept9-1a	F	GGCGGCACCATGAAGAAGT
	R	GAGTTGGGCGGCTCAATCT
Sept9-1b	F	ACCAGCCACCATGTCCGA
	R	GAGTTGGGCGGCTCAATCT
Sept9-1c	F	CAGGCAGCCCGACTTTCA
	R	GATCCCCACATAGCCGAACTC
Sept9-1d	F	CAGCCAGCAGAGCCCACTT
	R	GATCCCCACATAGCCGAACTC
Sept9-t	F	GGGCAGCGACCATGAGTATC
	R	GGAGATCCCGCAGGTAAGC
Ube2i-1a	F	GCCGCACAGGGGTTTCAG
	R	GGTCCTTCCTCCAGGCTTTC
Ube2i-1b	F	CTAAGTTCCGGGGATCGTCA
	R	GGCACAGGCTCTGGAGGTATC
Ube2i-1c	F	GGAGGGAAGTCCCGAGACAA
	R	GGTCCTTCCTCCAGGCTTTC

Biology Open • Supplementary information

Ube2i-t	F	TCATCCAAACGTGTATCCTTCTG
	R	CTTGTGCTCGGACCCTTTTCT
Uhrf1-1a	F	CTCACGCGGCTCCCTTCT
	R	TCTCCTTCCCATCCATAGTTCG
Uhrf1-1b	F	CCAGGGTCTGTGTCCCGAG
	R	TCTCCTTCCCATCCATAGTTCG
Uhrf1-t	F	CCACACCGTGAACTCTCTGTC
	R	GGCGCACATCATAATCGAAGA
Ubtf-1a	F	GCCCTCACTAGCACCCACTT
	R	CCGCTTCTCCGTTCATTCTC
Ubtf-1b	F	AGAGCCGAGAAGGGAGCC
	R	CCGCTTCTCCGTTCATTCTC
Ubtf-t	F	CCGCTGGTCCCAGGAAGATA
	R	CGACTCTGTGGTTTTGAACTTGG
Tjp2-1a	F	CTGGGACCGTCGCTTTCTG
	R	GCTCCCATATCACCTCCTCCA
Tjp2-1b	F	CAGCTTGTAGTTCTGAGCCGC
	R	GCTCCCATATCACCTCCTCCA
Tjp2-t	F	ATGGGAGCAGTACACCGTGA
	R	TGACCACCCTGTCATTTTCTTG
Pitx2-1a	F	ACCCTCAAGATCCCCGCAG
	R	AGAACGGCTGTCTCCCCGT
Pitx2-1b	F	CTAACACGGGGACACTTGGC
	R	CATGCAGTTCATGGACGAGG
Pitx2-t	F	ACCCCGGCTATTCGTACAAC
	R	GAGGACAGGGGATTGACGTTC
Net1-1a	F	ATCGGTCTCCTGGGCACTG
	R	ACCCTGCGGCTTCTCCTC
Net1-1b	F	TGCCCATTAAAAGGACGATCC
	R	TTTGCCAAAGATGTGACCCG

Biology Open • Supplementary information

Net1-t	F	AGCTCGGGTCACATCTTTGG
	R	CGGTGGACCTGCTGAATGA
Pdgfra-1a	F	GCTTGGGGCTTACTTTTCACTC
	R	CAGAAAGACCTGGTGGGAGGT
Pdgfra-1b	F	AGGCAGCAAAGAGGCAAGAT
	R	CAGAAAGACCTGGTGGGAGGT
Pdgfra-1c	F	GCTAGCGCGGAACCTCAGA
	R	CAGAAAGACCTGGTGGGAGGT
Pdgfra-t	F	ACAATAACGGGAGGCTGGTG
	R	AACTCGCTGGTCTTGAACGTC
Tjp1-e20 in	F	GAAGGACCACCATTGCCG
	R	CTGGGTGACCAAGAGCTGGT
Tjp1-e20 ex	F	AGCCTCTCAACAGGTGTACAGGA
	R	CTGGGTGACCAAGAGCTGGT
Tjp1-t	F	TTCCACCACCAGGCTTTACC
	R	TAGCCGAGGGCAGGACTTC
Epb41l5-1a	F	ACAATGGAGATAGGGCCAAAAG
	R	CTACCCCAGCCAGGGACAC
Epb41l5-1b	F	AAGAATGCAGGAGCCCATCA
	R	GTTCCACGGCCTAAAAGGTGT
Epb41l5-t	F	TGATGTCTCCTGTGCCTGTCAC
	R	TTCCTGTCGTGCTGCTCTGTC
Nf2-e16 in	F	TGAAACTCAAAGAGCGGGAGA
	R	TGAGGACTCAAATGCAGATAGGTC
Nf2-e16 ex	F	CGAGCTCAAGACGGAGATCG
	R	TGAGCTTTTTAATGGTATTATGCTTG
Nf2-t	F	GCCTGGCTCAAAATGGACA
	R	TCTCTTGAACTAGCTCCTCCTCAG
Fgfr2-IIIb	F	GTGGAAAAGAACGGCAGTAAATAC
	R	TTCCCCAGCATCCATCTCC

Biology Open • Supplementary information

Fgfr2-IIIc	F	GTGGAAAAGAACGGCAGTAAATAC
	R	TTCCCCAGCATCCTCAAAAG
Fgfr2-t	F	GCCTCTCGAACAGTATTCTCCT
	R	ACAGGGTTCATAAGGCATGGG
Rac1-e4 in	F	GCCTATGGGACACAGCTGGA
	R	TTGCCCCTGGAGGGTCTATC
Rac1-e4 ex	F	TGGAGAGTACATCCCCACCG
	R	AGAACACGTCTGTCTGCGGG
Rac1-t	F	AAGTGGTATCCTGAAGTGCGACA
	R	TGCGGGTAGGTGATGGGAG
Rbfox2-1a	F	CTCCCAAATAAACAGCCCTCC
	R	CGGTCCCTTGGTCCTTCAGT
Rbfox2-1b	F	TTCCAACTGGTTATCATCTGCTTC
	R	GAACGGGATGGTAGTAAAAGGCT
Rbfox2-t	F	CGCAATGGTTCAGCCTTTTAC
	R	CCGTAGAGGGTCAGGTTATGTTC
Gapdh	F	CCCCAATGTGTCCGTCGTG
	R	TGCCTGCTTCACCACCTTCT
